# Valorizing biomass into ultrahigh-surface-area porous carbon for sustainable next-generation energy storage

**DOI:** 10.1039/d5ra06592e

**Published:** 2026-02-26

**Authors:** Balanagouda N. Choudari, Shreeganesh Subraya Hegde, Mervin A. Herbert, Kumar G. N., Badekai Ramachandra Bhat

**Affiliations:** a Department of Mechanical Engineering, National Institute of Technology Karnataka Surathkal Mangalore 575025 Karnataka India; b Catalysis and Materials Chemistry Laboratory, Department of Chemistry, National Institute of Technology Karnataka Surathkal Mangalore 575025 Karnataka India hegdeshreeganesh@gmail.com ram@nitk.edu.in; c Department of Chemistry, School of Engineering, Dayananda Sagar University Horahalli Bengaluru Karnataka 562112 India

## Abstract

Developing biomass-derived porous carbon materials aligns with global efforts toward sustainable and environmentally friendly energy storage solutions. The renewable nature of this biomass-derived carbon material makes it well-suited for use in high-performance supercapacitor electrodes. This study employs a thermochemical activation method to synthesize ultrahigh-surface-area porous carbon from *Acacia auriculiformis* leaves. The synthesis process includes an initial pre-carbonization step followed by chemical activation, yielding a highly porous activated carbon with an exceptional BET surface area of 1913 m^2^ g^−1^. The electrochemical behavior of *Acacia auriculiformis* leaf-based activated carbon (ALC) electrodes was evaluated in a symmetric supercapacitor configuration with H_2_SO_4_ (1 M) as the electrolyte. At a current density of 0.5 A g^−1^, ALC electrodes achieved a specific capacitance (*C*_sp_) of 222 F g^−1^. The electrodes demonstrated robust cycling stability, retaining 89.3% of its initial capacitance even after 5000 cycles at a current density of 5 A g^−1^. This research highlights the viability of *Acacia auriculiformis* leaves waste as an abundant and renewable precursor for sustainable electrode materials, contributing to the advancement of eco-friendly energy storage technologies.

## Abstract 2

Schematic illustration of biomass-derived porous carbon materials for sustainable energy storage.

## Graphical abstract

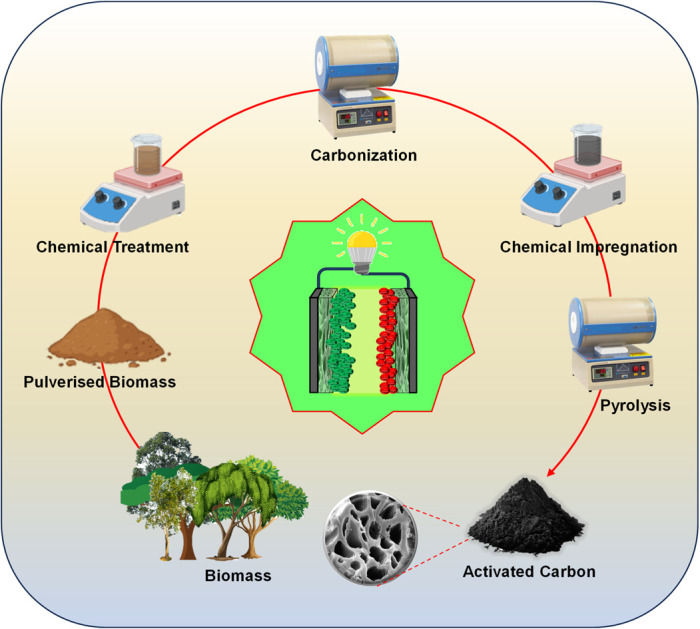

## 1. Introduction

In the 21^st^ century, sustainable development has become an urgent global priority to address climate change. One of the key drivers for sustainable development is the shift towards renewable energy sources.^1,2^ A surge in the adoption of renewable energy is driving a demand for the technologies that store and convert energy, including batteries, fuel cells, and supercapacitors.^3^ The demand for supercapacitors in energy storage technologies is growing rapidly, driven by their distinct advantages such as compact size, rapid charge–discharge capability, longer cycle life and favourable thermal stability. These characteristics make them particularly suitable for high-power-density applications, including renewable energy integration, electric vehicles, and portable electronic devices.^4^

There are different types of electrochemical capacitors based on their charge storage mechanism and active materials used. Electric double-layer capacitors (EDLC) use carbon-based active materials that store energy using ion adsorption, while pseudo-capacitors utilize fast and reversible surface reactions with redox-active materials. Hybrid capacitors, a new kind of EC, combine double-layer capacitive and pseudo-capacitive electrode materials, offering the benefits of both capacitors and batteries.^5^ Most commercially available supercapacitors employ carbon as the electrode material, due to its superior array of chemical and physical characteristics, including high electrical conductivity, a substantial specific surface area, excellent corrosion resistance, stability under thermal and chemical conditions, customizable pore structure, low cost, wide availability, and ease of processability.^6,7^

Materials derived from carbon, including graphene, carbon nanotubes, and activated carbon (AC) have become popular choices due to their promising properties.^8^ Among these carbonaceous materials, activated carbons derived from abundant biomass resources are easy to produce and cost-effective. Diverse biomass-based materials sourced from plants, agricultural waste, food waste, and biological-derived sources have undergone a conversion process, resulting in the production of activated carbon.^9^ Over the last few decades, plant waste-derived biomass has emerged as a promising precursor for producing carbon-based electrodes in energy storage devices. Because of their high carbon content and abundant availability, these natural resources serve as an excellent source for synthesizing carbon-rich materials efficiently.^10^ These carbon materials are designed to minimize environmental impact throughout their production, application, and disposal. Hence, they can be regarded as sustainable electrode materials for supercapacitors.^11^

In recent research studies, activated carbon is synthesised from various kinds of crop residue and biological materials. These precursors consist of agricultural byproducts and waste such as walnut shells, pig hair, mesua ferrea testa, onion flower seed cover & rice straw, date palm seeds, amygdalus davidiana shells, Japanese cedar bark, prosopis juliflora, cocoa pod husk, golden shower tree seed, *etc* as shown in Table 1. Among potential biomass resources, Acacia (*Acacia auriculiformis*) is one of the highly valued tree species in social forestry across tropical and subtropical regions. Its remarkable survival rate, rapid growth, and ability to form a dense canopy within a few years of planting have the capability of accumulating large quantities of biomass without requiring any agricultural input.^22^ Acacia leaves contain significant amounts of cellulose, hemicellulose, and lignin, which are organic compounds rich in carbon act as primary precursor for activated carbon.^23^ The leaves also contain several phytochemicals such as flavonoids, tannins, phenolic acids which may additionally improve surface chemistry and adsorption properties of the activated carbon.^24^ These components in the leaves make them highly suitable for conversion into activated carbon-based electrode materials for energy storage systems.

**Table 1:** Recent scientific quest for sustainable electrode materials in supercapacitors (SCs)

Biomass precursor	Remarks	Reference
Walnut shells	This study reports preparing AC from walnut shells by using self-activation strategy. The gases released during pyrolysis are used for activation. Outcome: *S*_BET_ = 1016.3 m^2^ g^−1^, *C*_sp_ = 146.9 F g^−1^ at 1 A g^−1^ (KOH, three electrode cell)	12
Pig hair	The influence of pre-carbonisation temperature (350 °C–500 °C) on capacitance was examined. And concluded higher the pre-carbonisation temperature better the structural disorder, specific surface area. PH500 activated with KOH at 800 °C performed better among others. Outcome: *S*_BET_ = 1002 m^2^ g^−1^, *C*_sp_ = 110 F g^−1^ at 5 mV s^−1^	13
Mesua ferrea testa	This study discussed about the effect of activation temperature on physiochemical properties of activated carbon. Material with activation temperature 700 °C delivered specific capacitance of 210 F g^−1^ at 1 A g^−1^ in an electrolyte of 3 M KOH. Cycle stability for material with 700 °C activation temperature was better than material with 800 °C when tested for 1000 cycles at 2 A g^−1^	14
Onion flower seed cover & rice straw	Activated carbon prepared from onion flower seed cover achieved 69% higher specific surface area than the other AC. Outcome: C_sp_ of 200.37 F g^−1^ in 1 M H_2_SO_4_ at 1 A g^−1^, cycle stability: 100% retention of capacitance (1000 cycles) at 6 A g^−1^	15
Date palm seeds	Date palm powder is mixed with NaC_2_O_4_, serving as chemical activation agent. Outcome: *S*_BET_ = 1630 m^2^ g^−1^, specific capacitance = 112 F g^−1^ at current density equal to 0.5 A g^−1^ and cyclic stability of 97% retention after 10 000 cycles	16
Amygdalus davidiana shells	Activated carbon prepared by mixing Amygdalus davidiana shells powder with ZnCl_2_. Outcome: *S*_BET_ = 508.90 m^2^ g^−1^, *C*_sp_ = 208 F g^−1^ at 1 A g^−1^ (three electrode system), 100% of initial capacitance after 20 000 cycles	17
Japanese cedar bark	This study used different chemical activators and found that KOH is best suited to produce high surface area. KOH activated carbon (KO-6) achieved specific capacitance of 210 F g^−1^ at 10 mA g^−1^ (1 M, H_2_SO_4_) and retained 80% starting capacitance over 6500 CV cycles at 1 mV s^−1^	18
Prosopis juliflora (Invasive weed)	This research focused on increasing oxygen functional groups by using KMnO_4_ as activator, and achieved O_2_ (36%). Among developed AC, JPF700 had 1210.4 m^2^ g^−1^ surface area and *C*_sp_ equal to 94 F g^−1^ @ 0.5 A g^−1^ in Na_2_SO_4_ (1M) electrolyte	19
Cocoa pod husk	The study focused deriving AC from different (500–800 °C) pre-carbonisation temperature. AC with 600 °C performed best SSA of 585 m^2^ g^−1^ and specific capacitance of 168 F g^−1^ at current density of 0.5 A g^−1^ in 2.5 M KNO_3_ electrolyte (two-electrode cell)	20
Golden shower tree seeds	This study prepares AC in two steps: first step includes carbonisation and chemical activation using potassium ferrate, second step involves modification of AC by anchoring with mesoporous carbon spheres derived from hard-template method. Outcome: *S*_BET_ = 1446 m^2^ g^−1^, *C*_sp_ = 154 F g^−1^ at 5 mV s^−1^ (KOH, three electrode cell) and *C*_sp_ = 69.5 F g^−1^ (1M LiPF_6_ electrolyte, two-electrode cell, 5 mV s^−1^)	21

This study presents a novel, cost-effective process for creating highly porous carbon, referred to as ALC, derived from *Acacia auriculiformis* leaves. The process follows a thermochemical approach to produce ultrahigh-surface-area porous carbon for sustainable supercapacitor electrodes. Characterisation techniques are employed to analyse the synthesised sample for physiochemical properties, while its electrochemical behaviour (cyclic voltammetry, GCD, EIS) is evaluated in a symmetric configuration.

## 2. Experimental section

### 2.1 Materials

Each chemical reagents utilised in this study was procured from reputed chemical suppliers. Loba Chemie Pvt. Ltd supplied potassium hydroxide (KOH) in pellet form, ethylenediaminetetraacetic acid disodium salt dihydrate (C_10_H_14_N_2_Na_2_O_8_·2H_2_O) and hydrochloric acid (HCl) for this study. Changshu Hongsheng Fine Chemicals supplied absolute ethanol (C_2_H_5_OH) which was used in synthesis. For electrode fabrication, the polymer binder (Polyvinylidene fluoride, PVDF) and the solvent (*N*-methyl-2-pyrrolidone, NMP) were both procured from Sigma-Aldrich. Commercial grade C-NERGY SUPER C65 (TIMCAL) and Grafoil sheets were used in their unaltered state. The ultrapure water (Type 1, Elga Veolia) was used to formulate all aqueous solutions. Naturally fallen and dried leaves of *Acacia auriculiformis* leaves (AL) were collected from the NITK Surathkal, Mangalore campus during dry season. The leaves were selected based on dryness, absence of contamination and decay. This biomass precursor was used to prepare *Acacia auriculiformis* leaf-based activated carbon (ALC).

### 2.2 Synthesis of *Acacia auriculiformis* leaf-based activated carbon (ALC)

Naturally fallen and dried *Acacia auriculiformis* leaves (AL) were collected, thoroughly washed, sun dried for a week before being pulverized. The preparation of high-surface carbon from AL was carried out using a thermochemical approach. Initially, AL powder was mixed with ethylenediaminetetraacetic acid disodium salt dihydrate in a 1 : 1 ratio using ultra-pure water. The mixture was initially heated to 150 °C under continuous stirring until a solid paste was formed. Subsequently, the dried mixture underwent carbonization in an argon atmosphere at 450 °C for 30 minutes. The resulting carbonized product was then treated with KOH (4 g) aqueous solution, and the suspension was stirred continuously on a hot plate at 150 °C to yield a uniform paste. This precursor was pyrolyzed in tube furnace at 700 °C for 30 minutes in an inert argon gas. Inorganic impurities were removed from the pyrolyzed samples by treating with a 30% HCl solution. Subsequently, the samples were subjected to multiple washing cycles using ultra-pure water to achieve neutral pH. The last synthesis step involved rinsing the samples with ethanol, followed by 24 hours drying period in an oven at 110 °C. The material obtained following this synthesis process was designated as ALC.

### 2.3 Characterizations

Using TGA 4000, thermogravimetric analysis was carried out by applying controlled heating ramp of 10 °C min^−1^ to measure the thermal stability of the material. Chemical functional groups present in material were identified using Fourier-transform infrared spectroscopy with a Spectrum 2 FTIR spectrometer (PerkinElmer, Singapore). The structural and morphologies of samples were characterized by following facilities: an Empyrean 3rd Gen diffractometer (Malvern PANalytical, Netherlands) was used to perform X-ray diffraction analysis using Cu-Kα monochromatic source with a characteristic wavelength (*λ*) equal to 0.154 nm; with a laser excitation wavelength of 532 nm, Raman analysis was undertaken using Confocal Raman microscope equipped with a Compact Raman spectrometer from Renishaw (UK); Energy dispersive X-ray and FESEM images were obtained using GEMINI 300, Carl Zeiss, Germany; The determine specific surface area, pore size, and pore volume N_2_ adsorption–desorption analysis was conducted on Autosorb IQ-XR-XR instrument (Anton Paar, Austria). Prior to measurement the samples after were outgassed under vacuum for 3 hours at 300 °C. X-ray photoelectron spectroscopy analysis was performed to understand the elemental composition of the sample.

### 2.4 Fabrication of electrodes and electrochemical characterizations

An electrode slurry containing ALC (80 wt%, active material), 10 wt% conductive carbon (C-NERGY SUPER C65), and 10 wt% of polyvinylidene fluoride (PVDF) was finely ground with a NMP solvent, was precisely drop-cast onto grafoil sheets (1 cm^2^) and vacuum dried at 60 °C. The resulting coating had 3 mg cm^−2^ as active material on substrate. The electrodes electrochemical performance was investigated within a symmetric electrode cell utilising a 1 M H_2_SO_4_ as the electrolyte. The electrochemical behavior of material was investigated using an electrochemical workstation (Autolab). Within a voltage range of 0–1 V, cyclic voltammetry was carried out at scan rates between 5–100 mV s^−1^. The same potential range was applied for conducting galvanostatic charge–discharge (GCD) tests at current densities starting from 0.5 A g^−1^ up to 3 A g^−1^. Electrochemical impedance spectroscopy (EIS) was measured in the frequency range of 0.01 Hz up to 1 MHz with a 10 mV amplitude. From the GCD data, key performance metrics such as specific capacitance (*C*_sp_, F g^−1^), energy density (*E*_d_, Wh kg^−1^), and power density (*P*_d_, W kg^−1^) were determined according to relationships mentioned below:1
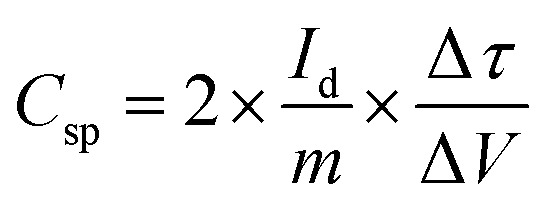
2
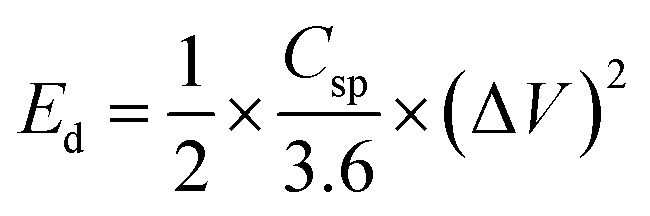
3
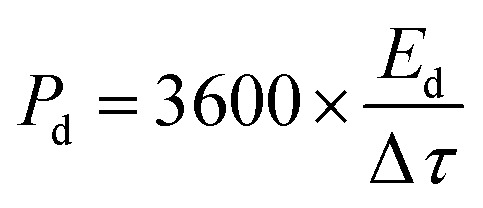
where, *I*_d_ is the discharge current in amperes (A), *m* is the mass of active material in grams (g), Δ*τ* is discharge time in seconds (*s*), Δ*V* is the potential change (*V*) within Δ*τ*.^25^

## 3. Results and discussions

### 3.1 Physicochemical and morphological characterizations

**Fig. 1:**
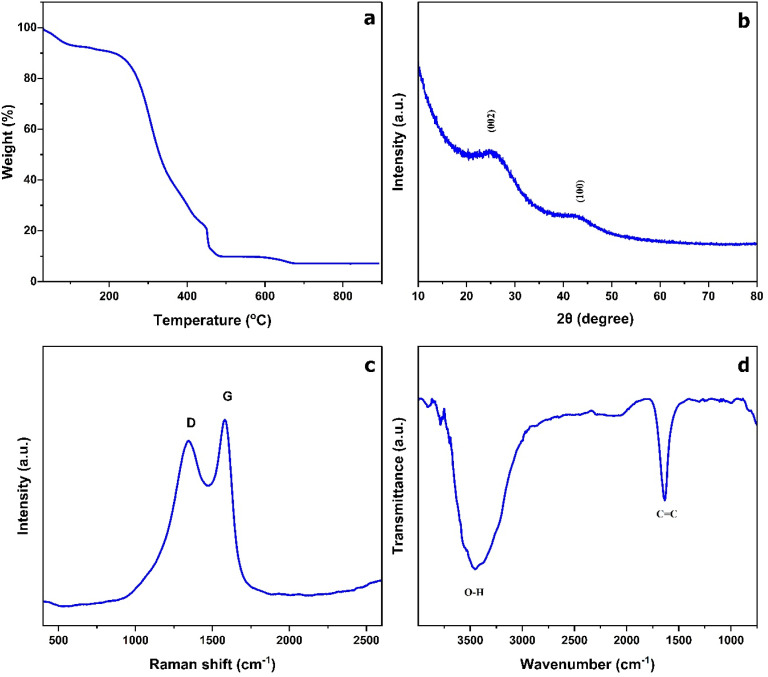
(a) TGA of AL powder; (b) XRD pattern (c) Raman spectroscopy (d) FTIR of ALC.

Thermogravimetric analysis (TGA) of AL powder was performed at a heating rate of 10 °C min^−1^ over a temperature range of 30 °C to 900 °C. The thermal degradation occurred in three stages, as shown in the TGA curve (Fig. 1a). In the initial stage, a mass loss at ∼100 °C was observed, corresponding to the evaporation of physically adsorbed moisture. This was followed by a pronounced thermal degradation between ∼250 °C and ∼450 °C, primarily associated with the decomposition of hemicellulose, cellulose, and partial breakdown of lignin.^26^ At this stage, blending with 2Na^+^ EDTA can influence the formation of pores and carbon structure, creating a significant increase in the surface area. In the third stage, more stable carbon structure is formed as the remaining lignin and other residual organic matter decompose over a wide temperature range.^27^ During this stage, the addition of potassium hydroxide as the activating agent can facilitate the penetration of the carbon matrix, which could result in the formation of a highly porous structure.

Fig. 1b displays XRD analysis, which reveals two prominent, broad diffraction peaks located at roughly 2*θ* ∼ 24° and 2*θ* ∼ 43° can be indexed to the (002) and (100) planes of carbon lattice respectively. The broadening of these peaks suggests that material has mainly amorphous carbon structure which is characterized by higher degree of structural disorder. The (002) peak is related to the stacking of graphene layers and points to the presence of turbostratic carbon. The interlayer spacing (*d*_002_) from (002) reflection calculated from Bragg's law (*nλ* = 2dsin *θ*) is 3.705 Å, which confirms disordered stacking.^28^ The characteristic features of carbon were also corroborated by Raman spectroscopy, showing two prominent peaks (Fig. 1c): the D-band at ∼1352 cm^−1^ which is induced by disorder and defects in the carbon matrix. Another peak at roughly 1594 cm^−1^ (G-band) induced by the tangential stretching of sp^2^ carbon bonds. The resulting intensity ratio (*I*_D_/*I*_G_ = 0.88) indicates a balance between disordered carbon and graphitic carbon.^29^

The surface functional composition of the sample is further evaluated by Fourier Transform Infrared (FTIR) spectroscopy analysis, as shown in Fig. 1d. The presence of hydroxyl functional groups is confirmed by a strong, broad absorption band at ∼ 3454 cm^−1^ which is a indicative of O–H stretching vibrations. The absorption peak at ∼ 1634 cm^−1^ is associated with the presence of C=C stretching vibrations in aromatic structures.^30^

**Fig. 2:**
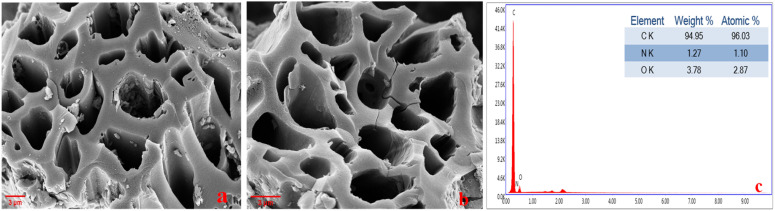
(a & b) FESEM (c) EDX of ALC.

Morphology also plays a key role in energy storage applications; therefore, the sample was analyzed using FESEM. Fig. 2a and b reveals the material's highly porous nature, resulting from a synthesis where EDTA was first mixed with precursor followed by successful carbonization and KOH chemical activation process. This order allowed EDTA to simultaneously improve nitrogen functionalization and initiate pore formation which was later expanded during chemical activation.^31,32^ This honeycomb inter connected porous architecture contributes to its specific surface area and ion diffusion pathways, which are essential for improving supercapacitor performance. In order to assess the material chemical composition, additional EDX analysis was performed; Fig. 2c, reveals the presence of carbon, oxygen, and nitrogen.

**Fig. 3:**
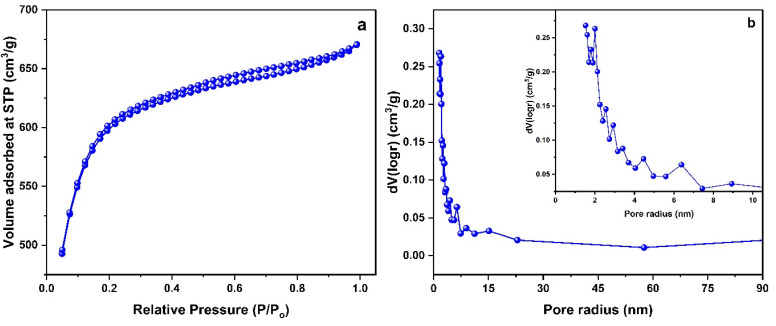
(a) N_2_ adsorption–desorption isotherm (b) pore size distribution curve of ALC.

The measurement of pore properties was conducted using nitrogen adsorption–desorption studies. The results shown in Fig. 3a indicate the type IV isotherm with hysteresis loop, which is characteristic of mesoporous materials. The initial sharp increase in adsorption volume at beginning of adsorption isotherm (*P*/*P*_0_ < 0.2) can be seen, which corresponds to microspores filling, implying the presence of microporosity in the material. The steady rise in adsorbed volume is observed between the relative pressure of *P*/*P*_0_ ∼0.2 up to 0.9 can be attributed to multilayer adsorption and capillary condensation occurring within materials mesopores. The relative pressures above ∼0.9 suggests existence of macropores.^33,34^ Pore size distribution curve as depicted in Fig. 3b, shows further evidence of materials hierarchical porosity. An impressively high BET surface area of 1913 m^2^ g^−1^ was accomplished due to the development of various types of pores during the chemical activation process.

**Fig. 4:**
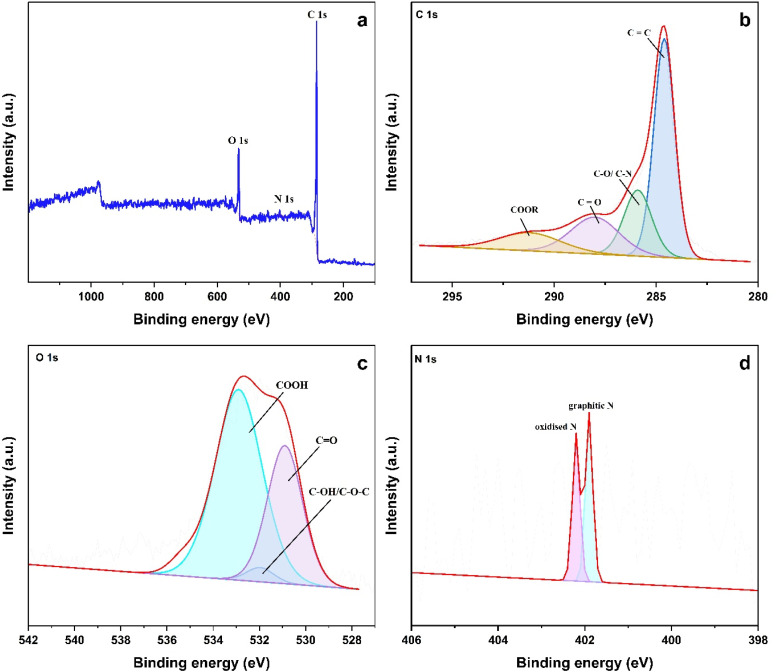
(a) XPS survey spectrum of ALC; XPS spectra of (b) C 1s (c) O 1s (d) N 1s of ALC.

In an attempt to find the surface chemical states of the ALC sample, X-ray photoelectron spectroscopy was performed afterwards. As shown in Fig. 4a carbon (C 1s), nitrogen (N 1s), and oxygen (O 1s) were clearly detected. The deconvolution of C 1s peak (Fig. 4b) indicates the presence of four distinct functional groups of carbon such as: sp^2^ carbon C=C bonds at 284.60 eV, C–N/C–O bonds at 285.90 eV, carbonyl groups C=O at 288.00 eV, and COOR groups at 291.20 eV.^35–37^ Likewise, O 1s was deconvoluted to yield three distinct peaks, as seen in Fig. 4c. These peaks corresponds to carbonyl (C=O at 530.90 eV), secondary alcohol or ether groups (C–OH or C–O–C at 531.97 eV), and the chemisorbed oxygen in a carboxylic groups accounts for peak at 532.90 eV.^38^ As shown in Fig. 4d, the high resolution of N 1s deconvolution revealed two nitrogen species: first peak at 401.9 eV is graphitic N and the second peak at 402.2 eV implies oxidised N.^39,40^ The presence graphitic N is known to contribute to the electrical conductivity thereby improving rate capability and cyclic stability, whereas oxidised N contributes to the wettability and pseudocapacitance.

### 3.2 Electrochemical performance

**Fig. 5:**
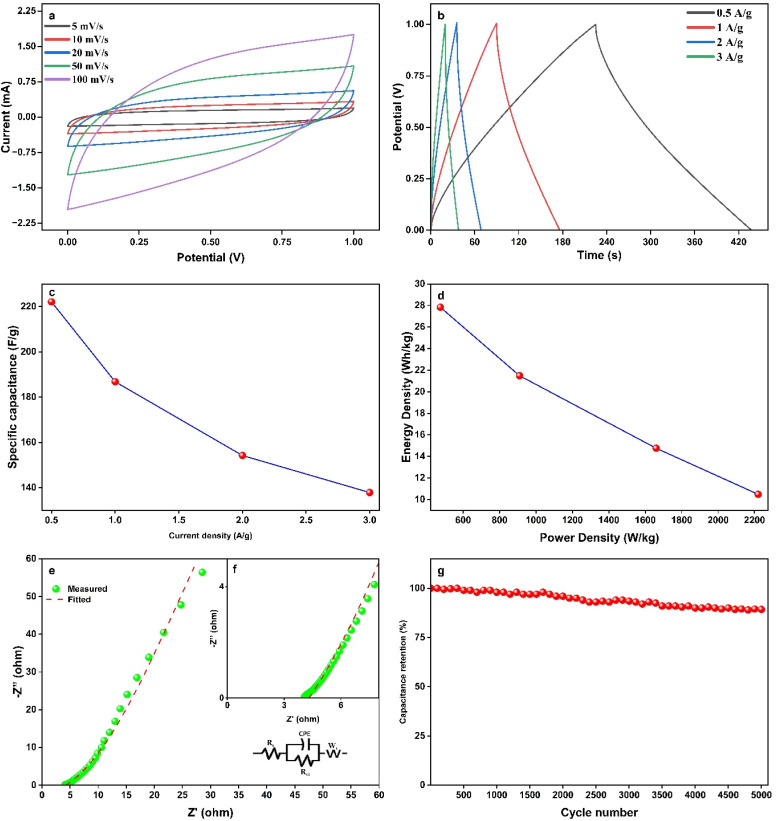
(a) CV, (b) GCD, (c) specific capacitance *v/s* current density, (d) Ragone Plot (e & f) EIS, (g) cyclic stability of ALC electrodes.

The electrochemical performance of ALC electrodes for supercapacitors was evaluated using a 1 M H_2_SO_4_ electrolyte. Fig. 5a reveals the quasi-rectangular appearance of cyclic voltammetry curves collected at scan rates spanning from 5 mV s^−1^ up to 100 mV s^−1^. This characteristic indicates charge storage mechanism that combines both electric double-layer capacitance and pseudocapacitive. The efficient charge storage in the ALC sample is attributed to dual mechanism: the large surface area promotes electric double-layer mechanism, whereas the presence of heteroatoms such as oxygen and nitrogen introduces additional pseudo-capacitance. Furthermore, the substantial area enclosed by the CV curves signified a high specific capacitance of the electrode materials.

Further confirmation was obtained through GCD studies conducted across various current densities, as illustrated in Fig. 5b. The GCD curves maintained nearly symmetrical profiles across all current densities, indicating excellent reversibility. At current density equal to 0.5 A g^−1^, ALC electrodes yielded high specific capacitance of 222 F g^−1^, this performance is credited to material's distorted honeycomb-like porous structure, which enhances ion transport. Additionally, the presence of heteroatoms such as oxygen and nitrogen enhances the energy storage mechanism by introducing pseudocapacitive behavior and improving electrochemical reactivity. Electrochemical performance of present study is compared with the existing biomass derived activated carbon-based supercapacitors as shown in Table 2. Fig. 5c illustrates reduction in specific capacitance is observed at higher current densities. This decline is a result of the rapid charge/discharge process at higher current densities, which limits the time available for electrolyte ions to diffuse into the deeper pores of the electrode material.^41,42^ As a result, only the outer surface actively contributes to charge storage, leading to a reduction in overall effective capacitance.^43^

**Table 2:** Comparative analysis between other existing biomass derived activated carbon electrodes in literature

Biomass precursor	Electrode configuration	Electrolyte	BET (m^2^ g^−1^)	Specific capacitance (F g^−1^)	Ref.
Ascophyllum nodosum (seaweed)	2E	1M H_2_SO_4_	1493	207.3 F g^−1^ at 0.5 A g^−1^	44
Sugar palm midrib	2E	1M H_2_SO_4_	557.641	210 F g^−1^ at 1 A g^−1^	45
Rice straw	3E	1M H_2_SO_4_	1502.01	120.46 at 1 A g^−1^	15
Onion flower seeds cover	2E	1M H_2_SO_4_	2538.31	158 F g^−1^ at 1 A g^−1^	
Amygdalus davidiana shells	2E	6M KOH	508.90	80.4 F g^−1^ at 1 A g^−1^	17
Waste-biomass tar	2E	6M KOH	592	199 F g^−1^ at 0.2 A g^−1^	46
Hazelnut seed shell	2E	1M H_2_SO_4_	—	166.2 F g^−1^ at 1 mV s^−1^	47
Acacia auriculiformis leaves	2E	1M H_2_SO_4_	1913	222 F g^−1^ at 0.5 A g^−1^	This work

Further, the energy and power densities of the ALC electrodes were calculated, and the corresponding Ragone plots, depicted in Fig. 5d, illustrate their superior electrochemical performance. At a power density of 475 W kg^−1^, the electrodes achieved a maximum energy density of 27.8 Wh kg^−1^. Electrochemical impedance spectroscopy was further used to evaluate electrodes. Nyquist plots in Fig. 5(e and f) reveal measurements for the ALC electrodes conducted over a frequency span of 0.01 Hz–1 MHz with a 10 mV amplitude. The EIS data was fitted to equivalent circuit model using ZSimp software to determine the resistive component values. The fitted parameters revealed *R*_s_ of 4.48 Ω and *R*_ct_ (charge transfer resistance) of 0.019 Ω. The plots elevated slope at low-frequencies suggests good capacitive behaviour, while its minimal resistance at the high-frequencies indicates fast electrochemical reaction kinetics in the ALC electrodes.^48^ The synergetic effect of several heteroatoms and large specific surface area are responsible for this performance.^49^ Finally, as shown in Fig. 5g, the long-term durability of ALC electrodes was examined through GCD testing at 5 A g^−1^. The electrodes revealed an outstanding retention of 89.3% of their starting specific capacitance even after completing 5000 cycles, highlighting their exceptional cycling stability and reliability. Therefore, these findings collectively validate the exceptional electrochemical performance of ALC materials, highlighting their potential for high-performance supercapacitor applications.

## 4. Conclusions

Developing biomass-derived porous carbon materials aligns with global sustainability initiatives, offering a viable pathway toward environmentally friendly and high-performance energy storage systems. In this study, ultrahigh-surface-area porous carbon was successfully synthesized from *Acacia auriculiformis* leaves using a thermochemical activation method. The porous carbon sample exhibited a significantly exceptionally large BET surface area of 1913 m^2^ g^−1^. Its well-developed hierarchical porous network, revealed through physiochemical and morphological characterisations promotes efficient ion transport and charge accumulation, thereby enhancing the electrochemical performance. At a current density of 0.5 A g^−1^, electrochemical measurements showed an outstanding specific capacitance of 222 F g^−1^. It also showed exceptional cycling stability and superior rate capability, maintaining 89.3% of its capacitance after 5000 charge–discharge cycles. These results open the door for scalable and environmentally friendly energy storage solutions by establishing ALC as a very effective and sustainable electrode material for supercapacitors.

## Conflicts of interest

There are no conflicts to declare.

## Data Availability

All the necessary and sufficient data supporting the findings of this study are provided in the manuscript. Any additional data relevant to the findings are available from the corresponding author upon reasonable request.
